# Comparison of Targeted Biopsy and Combined Biopsy to Avoid Unnecessary Systematic Biopsy in Patients with PI-RADS 5 Lesions

**DOI:** 10.3390/biomedicines11123163

**Published:** 2023-11-28

**Authors:** Changwei Yuan, Derun Li, Jingyun Wu, Qi Shen, Xiaoying Wang, Jiangxi Xiao, Zhisong He, Liqun Zhou, Xuesong Li, Yi Liu, Zheng Zhao

**Affiliations:** 1 Department of Urology, Peking University First Hospital, Institute of Urology, Peking University, National Urological Cancer Center, No. 8 Xishiku St., Beijing 100034, China; yuanpkufh@bjmu.edu.cn (C.Y.); ldr.kz.wayne@163.com (D.L.); 13522300373@163.com (Q.S.); hezhisong901@163.com (Z.H.); zhoulqmail@sina.com (L.Z.); pineneedle@sina.com (X.L.); 2 Department of Radiology, Peking University First Hospital, No. 8 Xishiku St., Beijing 100034, China; wujingyun1129@163.com (J.W.); cjr.wangxiaoying@vip.163.com (X.W.); cjr.xiaojiangxi@vip.163.com (J.X.)

**Keywords:** prostatic cancer, PI-RADS, combined biopsy, targeted biopsy, systematic biopsy

## Abstract

Purpose: To evaluate the detection rates of prostate cancer (PCa) and clinically significant prostate cancer (CSPCa) detection via target biopsy (TB), systematic biopsy (SB), and combined biopsy (CB) in patients with PI-RADS 5 lesions. Methods: Patients with at least one PI-RADS 5 lesion were retrospectively enrolled in a prospectively collected database. The patients underwent multiparametric magnetic resonance imaging (mpMRI) followed by transrectal TB of PI-RADS 5 lesions and SB. The PCa and CSPCa detection rates and cores of TB and SB were compared with those of CB. Results: In 585 patients, prostate biopsy revealed PCa in 560 cases (95.73%) and CSPCa in 549 cases (93.85%). PCa was detected in T2 patients (93.13%, 217/233) and in T3/4 patients (97.44%, 343/352). CSPCa was detected in T2 patients (89.27%, 208/233) and in T3/4 patients (96.87%, 341/352). The positive rates of TB for T2/3/4, T3/4, and T2 were 94.02%, 96.21%, and 90.56%, respectively. SB added 1.71% (10/585) PCa and 1.37% (8/585) CSPCa detection to TB. There was no difference between TB and SB in detecting different stages of cancer (*p* > 0.05). In the biopsy core analysis, TB had fewer biopsy cores and a higher detection rate than SB (all *p* < 0.05). Conclusions: In patients with PI-RADS score 5 lesions, TB can achieve the same detection rate as, with fewer biopsy cores than, CB. SB adds minimal clinical value and can be omitted for these patients.

## 1. Introduction

Prostate cancer (PCa) is the most frequently diagnosed malignancy in the male population and also constitutes the second leading cause of cancer-related death among men in Europe and America [1]. Multiparametric magnetic resonance imaging (mpMRI) as a noninvasive assessment tool has become a widely accepted imaging modality for PCa diagnosis and staging in clinical practice. The Prostate Imaging Reporting and Data System Version 2 (PI-RADS V2) provides clinical guidelines for the risk stratification of PCa and standard interpretation of mpMRI findings [2].

Prostate biopsy is paramount for establishing an efficient diagnosis. At present, systematic 12-core transrectal ultrasonography (TRUS) biopsy is commonly used for PCa diagnosis, but more biopsy cores undoubtedly cause more trauma. The use of MRI/ultrasound fusion to achieve targeted biopsy (TB) as a relatively new PCa diagnosis technology can guide puncture to specific suspicious lesions, showing higher accuracy and sensitivity compared with traditional TRUS-guided systematic biopsy (SB) [3,4]. A lesion with a PI-RADS score of 5 on mpMRI indicates clinically significant prostate cancer (CSPCa) with a detection rate of 80% [3]. Due to the high detection rate, the supplementary role of SB is uncertain. There is still a lack of evidence on whether TB can replace SB in detecting PI-RADS 5 lesions.

The present study aimed to investigate the detection rate of PCa through TB and SB to avoid unnecessary SB in patients with PI-RADS 5 lesions.

## 2. Materials and Methods

### 2.1. Study Population

We performed a retrospective review of patients undergoing ultrasound-guided prostate biopsy for PI-RADS 5 lesions in our hospital between January 2018 and June 2022. The inclusion criteria were as follows: (1) total prostate-specific antigen (tPSA) test before biopsy and clinical stage > T1; (2) transrectal or perineal prostate biopsy and pathological examination; and (3) PI-RADS score 5 on mpMRI. The exclusion criteria were as follows: (1) repeated biopsies; (2) incomplete clinical data; and (3) intolerance to biopsy. CSPCa in our study was defined as PCa with Gleason Score (GS) > 6 [5]. The combined biopsy (CB), i.e., TB + SB, was considered as the standard reference. The detection rates of PCa and CSPCa through TB and SB were compared. A total of 585 patients were enrolled in the study. The age, mpMRI, serum tPSA, and postoperative pathological results of patients were collected. TRUS was used to measure the prostate volume (PV). The f/tPSA and prostate-specific antigen density (PSAD) (tPSA/PV) were calculated. The clinical stage of PCa included both organ-confined PCa (T2) and locally advanced PCa (T3&T4). This study was approved by the Ethics Committee of Peking University First Hospital and informed consent was obtained from all patients.

### 2.2. MRI Examination

MRI was performed on a 3.0 T Discovery 750 MRI (GE Healthcare; Achieva, Philips Healthcare) with an 18-channel abdominal phased-array coil. Each patient was placed in a supine position, and the center of the scan was aligned approximately 2.0 cm above the pubic symphysis. mpMRI scanning sequence and parameters were based on PI-RADS V2 [2]. The scanning sequence included axial/sagittal/coronal T2WI, axial T1WI, diffusion-weighted imaging (DWI), and dynamic-contrast-enhanced (DCE)-MRI. After the completion of DWI, the apparent diffusion coefficient (ADC) was calculated and generated automatically by the post-processing software equipped with the scanner. Multi-phase dynamic contrast-enhanced DCE was performed with volume-fast 3D imaging sequence (LAVA or eTHRIVE sequence). The single-phase scanning time was 10 s, there were a total of 18 phases, and the dynamic scanning time was 180 s. The contrast agent gadolinium-diethylenetriamine pentaacetic acid (Gd-DTPA) was administered via the cubital vein. The mpMRI images of different sequences were uploaded to PACS system, and the main lesions were selected by two experienced and trained MR urologists based on T2WI, high B-value DWI, ADC, and/or DCE images. Tumor staging and PI-RADS scores were evaluated. The primary lesion was scored by the readers according to the criteria of PI-RADS v2.1 criteria (score 1—very low likelihood of CSPCa, score 2—low likelihood of CSPCa, score 3—undetermined, score 4—high likelihood of CSPCa, and score 5—very high likelihood of CSPCa). Any disagreement between the two doctors was resolved by consulting with the third radiologist. If the main lesion was located in the peripheral zone, DWI was used as the main scoring sequence. When DWI score was 5 points, which meant high B value with obvious focal high signal and ADC with obvious focal low signal, the maximum diameter was ≥1.5 cm, or there was extracapsular spread and the final score was 5 points. If the lesion was mainly located in the transitional zone, T2WI was used as the main scoring sequence, and a T2WI score of 5 was defined as a lens-shaped, unevenly bordered, homogeneous low signal, with a maximum diameter of ≥1.5 cm or extracapsular spread.

### 2.3. Prostate Biopsy

**Figure 1:**
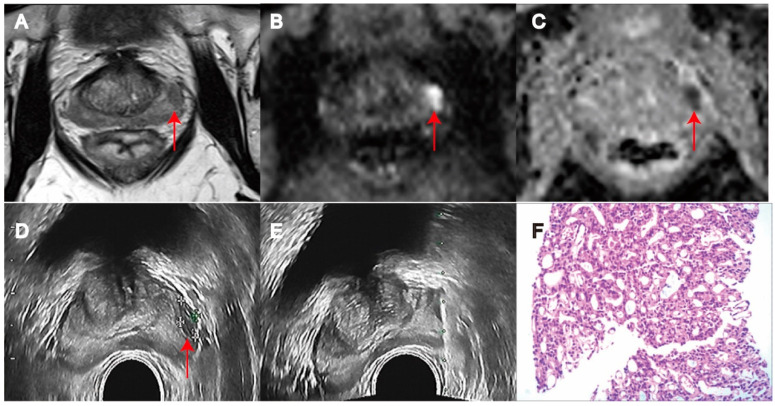
MRI/transrectal ultrasound fusion-guided transrectal target biopsy revealed Gleason 8 (Grade Group 4) prostate cancer in PI-RADS 5 lesion in peripheral transition zone of prostate (red arrow). (**A**) T2WI. (**B**) DWI. (**C**) ADC. (**D**) Transrectal ultrasound biopsy. (**E**) Target Biopsy. (**F**) PCa on pathology (H&E × 100).

**Figure 2:**
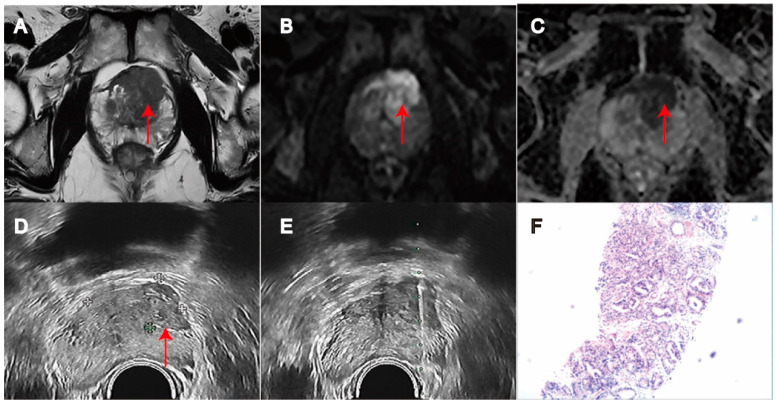
MRI/transrectal ultrasound fusion-guided transrectal target biopsy revealed Gleason 9 (Grade Group 5) prostate cancer in PI-RADS 5 lesion in transition zone of prostate (red arrow). (**A**) T2WI. (**B**) DWI. (**C**) ADC. (**D**) Transrectal ultrasound biopsy. (**E**) Target Biopsy. (**F**) PCa on pathology (H&E × 100).

The biopsy was performed by a urologist with experience in prostate biopsy. The ultrasound equipment adopted the color Doppler ultrasound diagnostic instrument (Hitachi, Philip). Prophylactic antibiotics were routinely used before surgery and one day before surgery. Each patient was placed in the left lateral position, and the prostate biopsy was performed under the guidance of a dual probe after routine disinfection and drape. The prostate volume was calculated using the following formula: volume (mL) = 0.52 * height (cm) * length (cm) * width (cm). Before the biopsy, urologists reviewed the suspicious lesions with a PI-RADS score of 5 on mpMRI under the guidance of radiologists, thereby making a definite diagnosis. The presences of focal hypoechoic lesions or contour bulges on gray-scale images were suspicious for malignancy. Power Doppler examination was also performed from the base to the apex, and focal asymmetric or increased blood flow density on power Doppler images was a suspicious sign for PCa (Figure 1 and Figure 2). For primary lesions, 1–3-core TB was performed, followed by 8–12-core SB. CB through four-zone 12-core biopsy was recommended by European Association of Urology (EAU) Guidelines, and patients with typical diffuse lesions on ultrasound underwent 6-core SB. Systematic and targeted biopsies were performed by the same physician. All specimens were individually labeled and analyzed by uropathologists who were blinded to imaging findings. The pathological evaluation included the number of positive cores, GS, and grade group (GG); these were evaluated and interpreted according to the recommendations of the International Society of Urological Pathology (ISUP) Grade Group. CSPCa in our study was defined as PCa with grade group (GG) > 2 or Gleason Score (GS) > 6.

### 2.4. Statistical Analysis

Statistical analysis was performed with SPSS software (IBM Corporation, Armonk, NY, USA, version 25.0). Categorical variables were shown as frequency values. Numerical variables were shown as mean ± standard deviation (m ± s) or interquartile range (IQR) values. Inter-group comparison was conducted using the Mann–Whitney U test for continuous data and Pearson’s chi-square test or Fisher’s exact test for categorical data. The paired chi-square test (McNemar test) was used to compare the detection rates of PCa and CSPCa through SB and TB. The coincidence of positive rate was applied to compare the positive rate of TB with that of CB. A value of *p* < 0.05 was considered statistically significant.

## 3. Results

CB is as regarded a gold standard for the detection of PCa with PI-RADS 5 lesions. The clinical and pathological characteristics of patients enrolled in this study are summarized in Table 1. In this cohort, the median (IQR) age was 70 years (64–76), tPSA was 25.42 ng/mL (13.09–73.65), PV was 49 mL (36.00–69.75), PSAD was 0.57 ng/mL^2^ (0.0.30–1.30), and lesion maximum size was 2.60 mm (1.90–3.55). In the T2 group, the median (IQR) age was 69 years (64–75), tPSA was 15.84 ng/mL (10.31–25.00), PV was 41.00 mL (30.05–54.80), PSAD was 0.39 ng/mL^2^ (0.23–0.65), and lesion maximum size was 2.00 mm (1.60–2.40). In the T3/4 group, the median (IQR) age was 70 years (65.00–76.75), tPSA was 45.82 ng/mL (19.24–130.50), PV was 57 mL (40.66–77.00), PSAD was 0.89 ng/mL^2^ (0.38–2.10), and lesion maximum size was 3.00 mm (2.43–4.10).

In 585 patients, prostate biopsy revealed PCa in 560 cases (95.73%) and CSPCa in 549 cases (93.85%). The cohort was assigned to the organ-confined group (T2) (39.83%, 233/585) and the locally advanced group (T3/4) (60.17%, 352/585). Statistical analysis comparing the T2 group and the T3/4 group showed that there were significant differences in age, tPSA, PV, PSAD, maximum diameter, and multiple lesions (all *p* < 0.05). The T3/4 group had a higher tPSA, larger PV, PSAD, and diameter than the T2 group. PCa was detected in T2 patients (93.13%, 217/233) and in T3/4 patients (97.44%, 343/352). CSPCa was detected in T2 patients (89.27%, 208/233) and in T3/4 patients (96.87%, 341/352). There were significant differences in the detection rates of PCa and CSPCa between the T2 group and the T3/4 group (*p* < 0.05). In these locally advanced PCa patients, as shown in Table 2, the median (IQR) age was 70 years (65.00–76.75), tPSA was 45.82 ng/mL (19.24–130.50), PV was 57 mL (40.66–77.00), PSAD was 0.89 ng/mL^2^ (0.0.38–2.10), and lesion maximum size was 3.00 mm (2.43–4.10). In the T3 group, the median (IQR) age was 71 years (64.75–77.00), tPSA was 35.52 ng/mL (15.45–25.00), PV was 41.00 mL (30.05–54.80), PSAD was 0.39 ng/mL^2^ (0.23–0.65), and lesion maximum size was 2.80 mm (2.00–3.50). In the T4 group, the median (IQR) age was 70 years (65.00–76.00), tPSA was 88.80 ng/mL (31.68–199.48), PV was 70 mL (55.00–95.03), PSAD was 1.20 ng/mL^2^ (0.46–3.16), and lesion maximum size was 4.00 mm (3.00–4.90). The tPSA, PV, PSAD, maximum diameter, and multiple lesions (all *p* < 0.05) had significant differences when it came to the T3 patients and T4 patients. The T4 group had a higher tPSA, larger PV, PSAD, and diameter. PCa was detected in T3 patients (96.73%, 207/214) and in T4 patients (98.55%, 136/138). CSPCa was detected in T3 patients (96.26%, 206/214) and in T3/4 patients (97.83%, 135/138). No significant difference was found between T3 and T4 patients in cancer detection rates (*p* > 0.05).

The biopsy results from TB, SB, and CB are shown in Table 3. The positive rate of TB for PCa and the positive coincidence between TB and CB were calculated. The positive rates of TB for T2/3/4, T3/4, and T2 were 94.02%, 96.21%, and 90.56%, respectively. The positive rates of coincidence between TB and CB were 98.21%, 98.83%, and 97.24%, respectively. For CSPCa, the positive rates of TB were 92.48%, 95.78%, and 87.55%, and the positive coincidence rates were 98.54%, 98.83%, and 98.08%. The positive rates of SB for T2/3/4, T3/4, and T2 were 95.38%, 96.88%, and 88.84%. For CSPCa, the positive rates of SB were 92.14%, 96.59%, and 85.41%, respectively. SB added 1.71% (10/585) PCa and 1.37% (8/585) CSPCa detection to TB in T2/3/4. SB added 2.58% PCa and 1.71% CSPCa in T2 and 1.14% PCa and 1.13% CSPCa in T3/4. The paired chi-square test (McNemar test) was applied to compare the detection rates of PCa and CSPCa through TB, SB, and CB (Table 3). The *p*-values were 0.832, 0.688, and 0.453 for PCa, respectively. The *p*-values were 0.815, 0.375, and 0.267 for CSPCa, respectively. There was no difference in the detection rates of PCa and CSPCa between TB and SB for PI-RADS 5 score patients (*p* > 0.05).

The numbers of cores and positive rates in prostate biopsy are shown in Table 4. The mean numbers of biopsy cores for CB, SB, and TB were 10.03, 8.73, and 2.14 (*p* < 0.05). CB had the most biopsy cores. The mean numbers of positive needles for CB, SB, and TB were 6.90, 4.98, and 1.92 (*p* < 0.05). And, the biopsy core positive rates for TB, CB, and SB were 0.90, 0.77, and 0.61 (*p* < 0.05). TB had the highest biopsy core positive rate.

## 4. Discussion

Prostate cancer is the most common cancer worldwide and the second leading cause of death among males in European and American countries [6]. Despite the remarkable achievements of detection methods including PSA, DRE, and mpMRI, prostate biopsy is still the gold standard for the diagnosis of PCa. There has been considerable concern regarding both the underdiagnosis of significant cancer and the overdiagnosis of clinically insignificant cancer with current biopsy strategies. EAU guidelines recommend TB + SB (12 cores) for prostate biopsy in the presence of suspicious lesions (PI-RADS score ≥ 3) on mpMRI. Studies have shown that PI-RADS 5 lesions are closely associated with CSPCa [3]. TB has a high diagnostic efficacy for CSPCa and the necessity of SB for supplementary diagnosis is still unclear. This study attempted to assess the feasibility of using TB alone without SB for prostate biopsy in patients with PI-RADS 5 lesions.

In the present study, the PCa and CSPCa detection rates on CB were 95.73% and 93.85%, higher than those reported in recent studies [7,8]. The presence of PI-RADS 5 lesions usually indicates a high probability of CSPCa, a large lesion volume, and a high proportion of locally advanced prostate cancer. The cohort was divided into the organ-confined group (T2) (39.83%) and locally advanced group (T3/4) (60.17%,). Firstly, there were significant differences in clinical characteristics between the T2 group and the T3/4 group. The locally advanced group had poor PCa variables as expected. The detection rates of PCa and CSPCa revealed significant differences between the T2 group and the T3/4 group (*p* < 0.05). The locally advanced group had a higher detection rate of both PCa (93.13% vs. 97.44%) and CSPCa (89.27% vs. 96.87%). Secondly, the patients with locally advanced PCa (T3/4) were further assigned to the T3 group and T4 group. Statistical analysis found no significant difference in cancer detection rates between T3 and T4. T2 patients had a lower tumor detection rate compared to T3/4 patients, which was in accordance with clinical observations and could be explained by the biological behavior of tumors. T2 tumors were generally limited to the organ or tissue, while T3 and T4 tumors had invaded beyond the organ or tissue boundary or metastasized to other sites. But, about the biopsy detection rates of PCa and CSPCa, there was no statistic difference between TB and SB in the same tumor stage. For PI-RADS 5 lesions, different cancer stages did not impact the patient’s biopsy scheme.

According to a paired cohort trial known as the Prospective Assessment of Image Registration in the Diagnosis of Prostate Cancer (PAIREDCAP) [9], TB is more efficient than SB in CSPCa detection, being most obvious in PI-RADS score 5 lesions. In the current study, CB was regarded as the gold standard. The detection efficiencies of TB and SB for different stages of cancer were compared, and the results revealed no difference in PCa and CSPCa detection rates between TB and SB, whether in the organ-confined group (T2) or the locally advanced group (T3/4). Some studies indicate that TB can obtain a similar detection efficiency to SB [8,10]. The positive rate detected through CB was very close to that detected through TB in patients with PI-RADS 5 lesions. For CSPCa, the positive rate of TB was 92.48% and that of CB was 93.85% in T2/3/4 patients. The positive coincidence rate for TB and SB reached above 97.24%, with a reliable confidence interval. SB only added 1.71% PCa and 1.37% CSPCa detection to TB. And, Tafuri et al. also found that systematic biopsy added limited cancer detection (2% PCa and 4% CSPCa) in 112 patients [8]. So, these studies demonstrated that TB achieved detection efficiency similar to CB in the absence of SB. Some scholars have also proposed that patients with high suspicious scores (like a PI-RADS score of 5) merely require TB [11]. In PI-RADS 5 patients, about 1/3 are localized prostate carcinoma patients from T2 and over 2/3 are T3/4 patients. Especially in patients with advanced T3 or T4 malignancies, surgical treatment is not recommended according to EAU guidelines. So, achieving a radical pathology is frequently not feasible. After the diagnosis has been confirmed through prostate biopsy, subsequently, the primary therapeutic interventions are usually radiotherapy, chemotherapy, and neoadjuvant endocrine therapy. Some studies had indicated that CB showed a similar rate of disease upgrading on radical prostatectomy in PI-RADS 5 patients and a significantly decreased upgrading rate in the PI-RADS 3–4 group, as compared to TB alone. The findings also showed that additional SB could be avoided in patients with PI-RADS 5 lesions, but that this is required for patients with PI-RADS 3–4 lesions [12].

However, some studies have shown that SB is still necessary for mpMRI suspicious lesions. Ahdoot et al. [13] believe that a combination of the two methods leads to more cancer diagnoses than with either method alone. If only TB is performed, 8.8% of clinically significant cancers will be misclassified. SB is still an important tool when evaluating all patients referred for prostate biopsy, but the demand for SB will decrease as the PI-RADS score increases. Arabi et al. [12] find that patients with PI-RADS 5 lesions may be safely managed with TB alone without combining SB. Patients with PI-RADS 3 and 4 lesions need SB in addition to MRI-TB for accurate management of their disease. Except for that, repeated SB is unlikely to uncover more aggressive PCa, particularly in the setting of a PI-RADS 5 lesion [10]. Tafuri et al. [8] demonstrated, in previous studies, that patients with PI-RADS 5 on mpMRI showed high positive predictive values for CSPCa upon prostate biopsy. In those patients with PSAD > 0.15 ng/mL^2^, SB marginally increases CSPCa detection but not overall PCa detection in comparison to TB alone. In this study, SB did not affect patients’ management and may be omitted for this population. These studies have suggested that patients with PI-RADS 5 lesions can be exempted from the potential redundancy of the SB.

The combination of TB and SB provides the most accurate and effective method for detecting PCa. However, the high accuracy of TB combined with SB comes at the cost of using more biopsy needles. The more prostate biopsy cores are involved in SB, the greater the risk of biopsy-related adverse events and complications such as infection, rectal bleeding, hematuria, hematospermia, erectile dysfunction, and retention will be [14,15]. Our results showed that TB with fewer than four to five biopsy cores had a higher positive rate than CB. This was consistent with the findings of Drobish et al. [10]. Only TB for PI-RADS 5 lesions can shorten the operation time and reduce patient discomfort, as well as the time to biopsy and specimen processing. Omitting SB can decrease the number of prostate biopsy cores, improve cost effectiveness and patient satisfaction, reduce the incidence of complications, and maintain adequate cancer detection rates.

The present study also had some limitations. First of all, since this study was a single-center retrospective study, there was some selection bias, inevitably. Second, some small lesions can be invisible in transrectal ultrasound-guided biopsies. Then, we used CB as the reference standard, which introduced the risk of false-negative diagnosis. Finally, all biopsies were performed by two experienced urologists, and the current investigation was performed by radiologists with more than ten years of experience. However, there is a strong subjectivity in the selection of suspicious areas and targeted puncture of suspicious areas, highly depending on the experience of the puncture operator, which may affect the accuracy of a targeted puncture. We are optimistic that this study can serve as a foundation for developing a more effective puncture scheme for PCa. A larger number of studies are needed to confirm these results and achieve further evidence about the clinical effects.

## 5. Conclusions

In patients with PI-RADS 5 lesions, TB can obtain the same detection rate with fewer prostate biopsy cores when compared to CB. SB adds minimal clinical value and can be omitted for these patients. However, a prospective and randomized study with a larger sample size is warranted to confirm the value of the biopsy scheme.

## Figures and Tables

**Table 1:** Demographics and clinical characteristic of patients with PI-RADS 5.

PI-RADS 5	N = 585	T2 (233)	T3/4 (352)	*p*-Value
Age, yr	70.00 (64.00,76.00)	69.00 (64.00, 75.00)	70.00 (65.00, 76.75)	0.021
tPSA	25.42 (13.09, 73.65)	15.84 (10.31, 25.00)	45.82 (19.24, 130.50)	0.000
PV	49.00 (36.00, 69.75)	41.00 (30.05, 54.80)	57.00 (40.66, 77.00)	0.000
PSAD	0.57 (0.30, 1.30)	0.39 (0.23, 0.65)	0.89 (0.38, 2.10)	0.000
Maximum diameter	2.60 (1.90, 3.55)	2.00(1.60, 2.40)	3.00 (2.43, 4.10)	0.000
lesions				
Multiple	422 (72.14%)	135	287	0.000
Single	163 (27.86%)	98	65	
PCa. %	95.73	93.13	97.44	0.012
csPCa. %	93.85	89.27	96.87	0.000

PCa: prostate cancer; CSPCa: clinically significant prostate cancer; PV: prostate volume; PSAD: prostate-specific antigen density.

**Table 2:** Comparison of demographics of patients with locally advanced PCa.

PI-RADS 5	N = 352	T3 (214)	T4 (138)	*p*-Value
Age, yr	70.00 (65.00, 76.75)	71.00 (64.75, 77.00)	70.00 (65.00–76.00)	0.852
tPSA	45.82 (19.24, 130.50)	35.52 (15.45, 80.52)	88.80 (31.68, 199.48)	0.000
PV	57.00 (40.66, 77.00)	47.32 (36.00, 65.00)	70.00 (55.00–95.03)	0.000
PSAD	0.89 (0.38, 2.10)	0.74 (0.36, 1.52)	1.20 (0.46, 3.16)	0.006
Maximum diameter	3.00 (2.43, 4.10)	2.80 (2.00, 3.50)	4.00 (3.00, 4.90)	0.000
lesions				0.000
Multiple	287 (81.53%)	159	128	
Single	65 (18.47%)	55	10	
PCa. %	97.44%	96.73	98.55	0.491
csPCa. %	96.87%	96.26	97.83	0.538

PCa: prostate cancer; CSPCa: clinically significant prostate cancer; PV: prostate volume; PSAD: prostate-specific antigen density.

**Table 3:** Positive rate comparison of TB and CB.

PI-RADS 5	CB (Gold Standard)	TB	TB Positive Coincidence Rate	95%CI	SB Added Value	TB	SB	McNemar *p*-Value
PCa								
T2/3/4	95.73	94.02	98.21	97.14–99.29	1.71	94.02	95.38	0.832
T3/4	97.44	96.21	98.83	97.71–99.96	1.14	96.21	96.88	0.688
T2	93.13	90.56	97.24	95.13–99.34	2.58	90.56	88.84	0.453
CSPCa								
T2/3/4	93.85	92.48	98.54	97.57–99.51	1.37	92.48	92.14	0.815
T3/4	96.87	95.74	98.83	97.70–99.95	1.13	95.74	96.59	0.375
T2	89.27	87.55	98.08	96.31–99.84	1.71	87.55	85.41	0.267

PCa: prostate cancer; CSPCa: clinically significant prostate cancer; CB: combined biopsy; TB: detection on target biopsy; SB systematic biopsy.

**Table 4:** Number of cores and positive rate in prostate biopsy.

PI-RADS 5	CB	TB	SB	*p*-Value
number of biopsy cores	10.03 ± 3.63	2.14 ± 0.77	8.73 ± 3.05	0.000
number of positive cores	6.90 ± 3.09	1.92 ± 0.83	4.98 ± 2.84	0.000
Positive rate	0.77 ± 0.40	0.90 ± 0.26	0.61 ± 0.32	0.000

CB: combined biopsy; TB: detection on target biopsy; SB: systematic biopsy.

## Data Availability

The datasets generated during and/or analyzed during the current study are available from the corresponding author on reasonable request.
